# Before considering immunoglobulins for Guillain-Barré syndrome as a cause of false-positive hepatitis serology, all other explanations should be ruled out!

**DOI:** 10.11604/pamj.2025.52.183.50441

**Published:** 2025-12-24

**Authors:** Josef Finsterer

**Affiliations:** 1 Neurology Department, Neurology and Neurophysiology Center, Vienna, Austria

**Keywords:** Guillain-Barré syndrome, immunoglobulins, hepatitis B serology, false positive result, HBs antibodies

## Abstract

A false-positive hepatitis serology result may not be solely due to intravenous immunoglobulins (IVIG) administration but may also result from cross-reacting antibodies from other infections, autoimmune diseases, recent hepatitis B vaccination, or interactions with rheumatoid factor or biotin. Furthermore, technical errors, such as faulty reagents or unsuitable testing methods, as well as certain patient groups, for example, those with certain chronic diseases, can also contribute to false-positive results. More likely than interference from IVIGs, the false-positive hepatitis serology result in one of the studies we read is due to Henoch-Schönlein pupura.

## Reaction

We read with interest the article by Bendimrad *et al*. about a 63-year-old Moroccan man with Guillain-Barré syndrome (GBS) and Henoch-Schönlein purpura (HSP). After treatment with intravenous immunoglobulin (IVIG), he developed false-positive anti-HBs and anti-HBc antibodies [1]. A negative hepatitis B virus DNA PCR test confirmed the absence of an active infection [1]. The IVIG was identified as the cause of the false-positive hepatitis B serology [1]. The study is interesting, but some points require discussion.

First, a false-positive hepatitis serology result may not be solely due to IVIG administration but may have other causes. For example, cross-reacting antibodies from other infections, autoimmune diseases, recent hepatitis B vaccination, or interactions with rheumatoid factor or biotin can lead to a false-positive result [2]. Technical errors, such as faulty reagents or unsuitable testing methods, as well as certain patient groups, for example, those with certain chronic diseases, can also contribute to false-positive results. As long as these alternative causes have not been completely ruled out, IVIG cannot be held responsible for the false-positive hepatitis serology result. More likely than interference from IVIGs, the false-positive hepatitis serology result in the index patient is due to HSP. HSP can cause false-positive hepatitis serology results because some hepatitis infections, particularly hepatitis B, can trigger the same type of immune complex reaction that causes HSP. In these cases, the immune system forms immune complexes containing hepatitis antigens and antibodies, which can then lead to a positive hepatitis test without current or active infection [3].

Second, IVIGs can temporarily cause false-positive results for both hepatitis B surface antibodies (HBs) and hepatitis B core antibodies (HBc). This is due to the passive transfer of antibodies from the pooled plasma of donors [4]. This phenomenon is well documented and must be recognised to avoid misinterpretations of a patient's hepatitis B status and potentially unnecessary interventions. The batch used to treat the index patient should therefore be tested for hepatitis antibodies.

Third, the index patient was discharged after IVIG administration and may have come into contact with numerous infectious agents at home or in public, which could have triggered the production of hepatitis antibodies.

The fourth point concerns infection via IVIGs, which can be ruled out solely based on the timeline of antibody development. HBs antibodies develop approximately one week after infection, while HBc antibodies develop no earlier than five weeks later. Since the index patient already showed positive HBc antibodies two weeks after IVIG administration, these could not have originated from the IVIGs if they carried the hepatitis B virus, which was clearly not the case.

The conclusion that IVIGs can influence viral serological tests - a factor that should be considered to avoid misinterpretations and unnecessary interventions - remains questionable until all alternative causes, particularly HSP and the transfer of antibodies from the IVIGs to the patient, have been ruled out.
